# Impact of covid 19 outbreak on mental health of patients with cancer

**DOI:** 10.1192/j.eurpsy.2022.1282

**Published:** 2022-09-01

**Authors:** W. Sbika, H. Belfekih

**Affiliations:** Mohamed Taher Maamouri university Hospital, Department Of Medical Oncology, Nabeul, Tunisia

**Keywords:** cancer, mental health, COVID 19 outbreak, depression and anxiety

## Abstract

**Introduction:**

The COVID19 outbreak was declared a public health emergency by The Word Health Organisation (WHO) on January 2020. By spring 2020, more than half of the world’s population had experienced a lockdown with strict pandemic prevention such as physical distancing measures. The COVID-19 pandemic have negatively affected many people’s mental health especially the ones who are at risk such as patients with cancer.

**Objectives:**

This study aimed to screen mental health problems among patients with cancer during the fisrt wave of COVID 19.

**Methods:**

To assess the impact of COVID-19 outbreak on mental health of patients with cancer, a Survey was conducted at the department of medical oncology in Nabeul (Tunisia) between March and May 2020. The patients were asked to answer a socio-demographic questionnaire. The COVID-19 infection-related mental Heath problems were measured using the **H**ospital **A**nxiety and **D**epression **S**cale (HADS). Medical conditions and clinical characteristics were extracted from patients healthcare records.

**Results:**

The median age was 53 years (range, 34-70) with sex ratio 0.35. The majority of the patients had a social support (85%) and lived in urban areas (60%). Only 19 % of them had college degree. Almost quarter of patients had medical conditions. The most common cancer in our cohort was breast cancer (54%) followed by colorectal cancer (20%). Sixty four per cent of them were on adjuvant chemotherapy. Among the 80 person surveyed, 20% had depression and 39 % anxiety.

**Conclusions:**

Further investigations are required to screen mental health status for all cancer patients in order to help them coping.

**Disclosure:**

No significant relationships.

